# Creutzfeldt-Jakob Disease in a Saudi Female: A Case Report

**DOI:** 10.7759/cureus.75887

**Published:** 2024-12-17

**Authors:** Fahad Albadr, Saba M Aldusaymani, Yousef A Aldobikhi, Sanad I Alkhaldi, Hatim S Sendy, Hamdan S Aldosari, Abdulaziz S Aljurayyad

**Affiliations:** 1 Radiology and Medical Imaging, King Saud Medical City, Riyadh, SAU; 2 Neuroradiology, King Saud University, Riyadh, SAU; 3 Medicine, AlMaarefa University, Riyadh, SAU; 4 Department of Diagnostic Radiology, King Khalid University Hospital, Riyadh, SAU; 5 Medicine, Northern Border University, Arar, SAU; 6 Medicine, Imam Mohammed Ibn Saud University, Riyadh, SAU; 7 Medicine, King Saud University Medical School, Riyadh, SAU

**Keywords:** clinical case report, creutzfeldt-jakob disease, kingdom of saudi arabia (ksa), neurodegenerative disorder, progressive dementia

## Abstract

Creutzfeldt-Jakob disease (CJD) is a rare, rapidly progressive, and incurable neurodegenerative disorder caused by prions. It is invariably fatal and classified under transmissible spongiform encephalopathies. This case report presents a 66-year-old Saudi female who was admitted to the neurology department due to a rapidly advancing cognitive decline. The patient underwent diagnostic evaluation, including magnetic resonance imaging (MRI) and electroencephalogram (EEG). Following a month of hospitalization with psychosocial support, the patient was stable and subsequently discharged. In conclusion, while CJD is an uncommon condition, it should be considered in the differential diagnosis of patients presenting with rapidly progressive dementia. Early and accurate diagnosis is essential to differentiate this untreatable disease from other treatable forms of rapidly progressive dementia and to facilitate potential future therapeutic interventions.

## Introduction

The rare neurological disorder known as Creutzfeldt-Jakob disease (CJD) is a transmissible, deadly disease with an accelerated course of illness caused by prion proteins, which are typical neuron proteins mainly composed of α-helical and random coil structures. Proteinaceous infectious particles, are self-propagating proteins lacking nucleic acid and are mostly compromised of proteinase K-resistant β-pleated sheet aggregates. Prions replicate by binding with normal prion cellular isoforms and changing α-helices into indigestible β-pleated sheets. These particles lead to the development of CJD and other transmissible spongiform encephalopathies [1]. There are four known subtypes of Creutzfeldt-Jakob disease: sporadic, variant, familial, and iatrogenic. The rate of occurrence of the disease is 1/1,000,000 per year with an average age of onset at 60 years old [2]. The disease mainly affects the central nervous system leading to damage in the neurons commonly manifesting as progressive dementia, visual changes, myoclonus, and ataxia. Some unusual symptoms may also present as chorea, psychiatric symptoms, and sleep disturbances as well as peripheral neuropathies. The diagnosis of Creutzfeldt-Jakob disease is a difficult task. With brain biopsy being the best method of diagnosis by histopathological confirmation of the disease, less invasive means of diagnosis along with the clinical picture are imaging, biomarkers, and electroencephalography [3]. This paper will report a case of a Creutzfeldt-Jakob in a 66-year-old Saudi female.

## Case presentation

A 66-year-old Saudi female presented to the Emergency Department with rapidly progressive dementia. She was in a normal mental state of health before contracting COVID-19 pneumonia on 29/08/2021. After that, she became depressed with excessive crying and reduced social interest, with fluctuating memory loss. During the previous five months, the problem got worse. She became more forgetful and disoriented, alienated her family, and experienced hallucinations and delusions of persecution. Her inability to understand her family, especially during the last month, limited assessment of cognitive function due to severe comprehensive impairment. She can follow simple commands. There were occasional mild tremors in her right hand. There was no history of loss of consciousness, weakness, gait impairment, numbness, falls, bulbar symptoms, headache, dizziness, or any other neurological symptoms, and no previous history or family history of similar complaints. She had no known surgical history and is a known case of unmedicated hypothyroidism (thyroid-stimulating hormone (TSH): 5.11, free thyroxine (FT4): 16). She was electively admitted to the neurology department for one month and was vitally stable. Vitals were temperature of 36.7 C, heart rate 78, respiratory rate 19, blood pressure 120/64, and oxygen saturation (SpO2) 97%. A neurological examination was done to the best of the patient’s ability and it showed severe limitation in cognition and comprehension with Parkinsonian features. Motor examination was not properly tested as her comprehension was severely impaired, however, the patient was able to move all of her limbs with no asymmetry, and symmetrical brisk reflexes (3+) except the triceps, which was 2+. Cranial nerve examination was normal, and sensory and cerebellar signs were not tested. 

**Figure 1:**
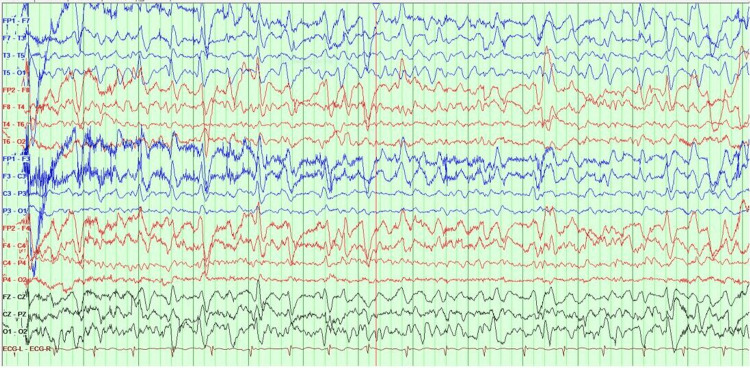
Abnormal EEG during wakefulness and sleep which demonstrated generalized slowing and generalized triphasic periodic discharges. These findings can be seen in sporadic Creutzfeldt-Jakob Disease (SCJD), medication toxicity and Alzheimer's disease. Moreover it is considered an ictal and interictal continuum (possible electrographic seizure).

**Figure 2:**
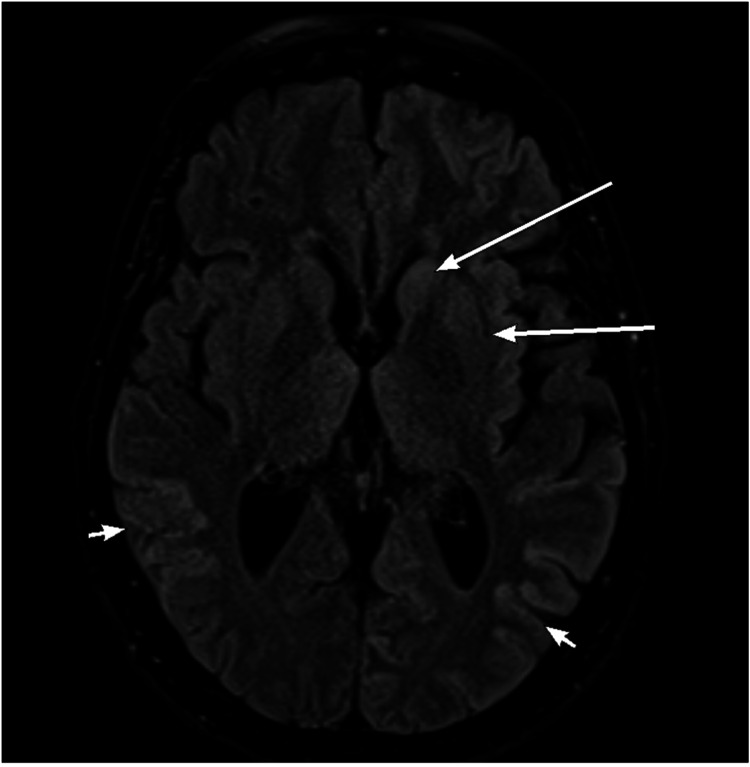
Fluid-attenuated inversion recovery (FLAIR) image of the brain showing subtle hyperintensity involving the parietal cortex bilaterally with an asymmetric pattern (small arrows), also involving the left caudate head and left putamen (long arrows).

**Figure 3:**
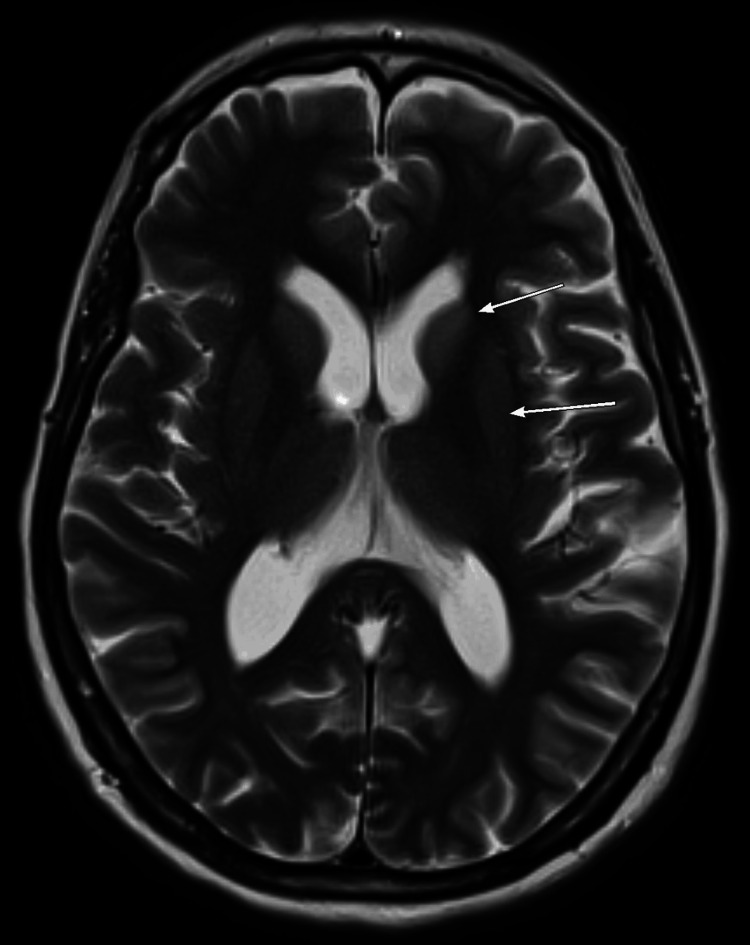
T2-weighted image showing signal alteration of the aforementioned deep gray matter (large arrows).

**Figure 4:**
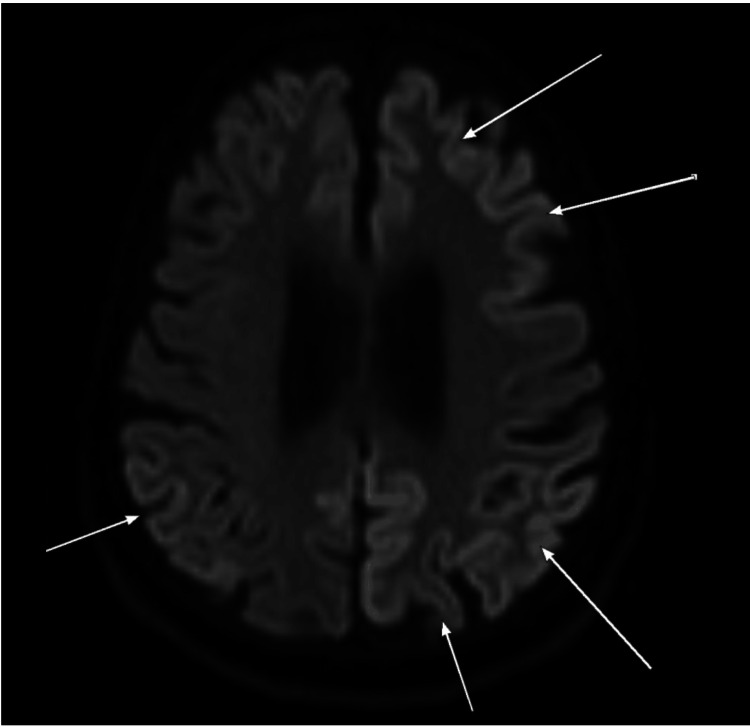
Diffusion-weighted imaging (DWI) demonstrating diffusion restriction characterized by cortical ribboning in the left frontal and bilateral parietal lobe cortices (long arrow).

**Figure 5:**
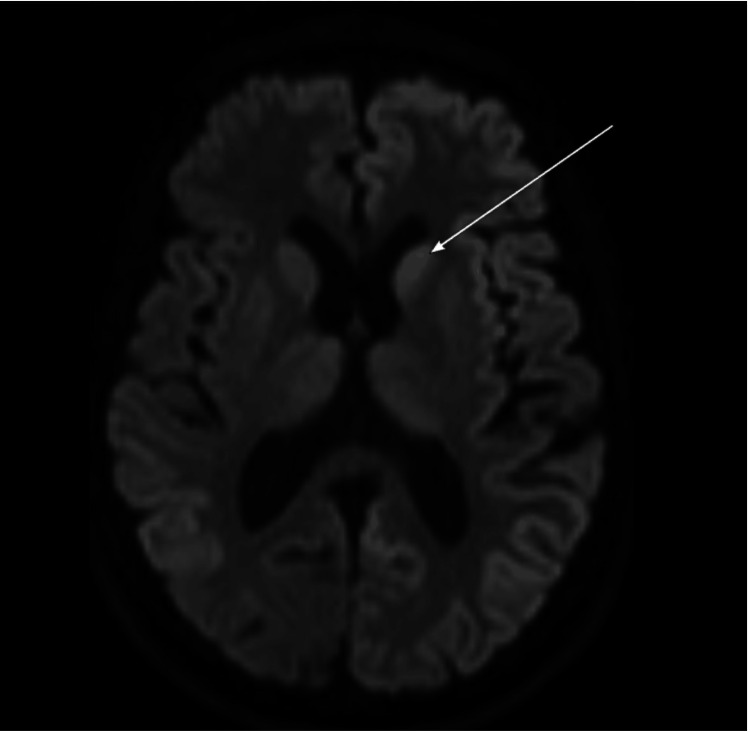
Diffusion-weighted imaging (DWI) showing diffusion restriction of the left caudate head (long arrow).

Electroencephalogram (EEG) showed an abnormality during wakefulness and sleep which demonstrated generalized slowing and generalized triphasic periodic discharges (Figure 1). A lumbar puncture for cerebrospinal fluid (CSF) examination was done. The CSF showed many RBCs, rare mature lymphocytes, and no abnormal cells. However, CSF analysis for 14-3-3 protein identification was not done. The patient was stable with no active complaints, and she was discharged on psychosocial support. Magnetic Resonance Imaging (MRI) with contrast was done (Figures 2-5). 

## Discussion

Creutzfeldt-Jakob disease is a rare neurodegenerative brain disorder of unknown etiology. It has a rapidly worsening course and fatal outcome. Most patients die within one year of the onset of manifestations [4].

This patient was diagnosed with sporadic CJD based on her presentation with neuropsychological manifestations mainly in the form of rapidly progressive dementia, severely impaired cognition, and comprehension with hallucinations and delusions, along with the suggestive brain MRI findings and EEG features.

The present case was a female aged 66 years. In this regard, Rus et al. [5] reported a case series of nine CJD patients who were mostly females, with a mean age of 71.6±10.0 years. Furthermore, Mackenzie and Will [6] reported that sporadic CJD typically affects patients in the seventh decade of life, with a lower incidence in the age group of 20-40 or older than 80.

Early diagnosis of CJD is always challenging due to the wide spectrum of symptoms of the disease which are similar to other forms of dementia [5]. Patients usually present with non-specific behavioral abnormalities including depressive symptoms, anxiety, loss of appetite, reduced social interest, change in sleep pattern, fatigue, and confusion. Furthermore, most of these patients suffer from amnesia and cognitive impairment which become severe with disease progression. Advanced cases may show other neurological disorders such as aphasia or apraxia, choreiform-athetoid movements, myoclonus jerks, and pyramidal or extrapyramidal signs [7,8].

According to the European criteria for diagnosing CJD, a diagnosis of probable CJD is based on rapidly progressive dementia in conjunction with one or more of the following clinical manifestations: myoclonus, pyramidal or extrapyramidal dysfunction, disordered visual or cerebellar function, and akinetic mutism. A diagnosis of possible CJD requires the presence of rapidly progressive dementia but lacks the additional clinical symptoms necessary for a probable diagnosis. Confirmation of the diagnosis for both probable and possible CJD may be supported by findings such as typical periodic sharp wave complexes on EEG, elevated levels of the protein 14-3-3 in cerebrospinal fluid, or MRI hyperintensity in the putamen and caudate nucleus or at least two cortical regions observed in either fluid-attenuated inversion recovery (FLAIR) or diffusion-weighted imaging (DWI) sequences [9].

In contrast, the U.S. Centers for Disease Control and Prevention (CDC) criteria classify CJD cases into "definite," "probable," and "possible." For a definitive diagnosis, the CDC requires the presence of a characteristic prion protein accumulation in brain tissue, usually confirmed through brain biopsy or autopsy. A probable diagnosis by CDC standards includes rapidly progressive dementia, along with at least two of the following clinical features: myoclonus, visual disturbances, ataxia, or pyramidal/extrapyramidal dysfunction, in the absence of other causes. Possible CJD according to the CDC needs the presence of rapidly progressive dementia along with at least one of the aforementioned clinical features but lacks sufficient diagnostic studies or definitive confirmation [9].

The patient’s brain MRI showed altered signal intensity in different parts of the cerebral cortex of asymmetrical pattern, with concomitant diffusion resection within the deep gray matter, especially in the left caudate head and medial aspect of the thalami. Similarly, Salehian et al. [10] have reported hypersignality in caudate, and putamen areas in a case of sporadic CJD. In addition, Mackenzie and Will [6] have identified brain MRI as an important non-invasive tool that could accurately recognize all CJD forms. They reported that high-signal patterns in the caudate, putamen, or cortex (or a combination of these) are essential findings in patients with sporadic CJD. In addition, Caobelli et al. [11] showed that brain MRI is used extensively in the diagnosis of CJD patients, and its diagnostic accuracy is higher than other neuroimaging techniques such as positron emission tomography (PET), and single-photon emission tomography (SPECT).

The EEG study of this patient showed an abnormality during wakefulness and sleep which demonstrated generalized slowing and generalized triphasic periodic discharges. Periodic sharp wave complexes are the most commonly reported EEG findings in CJD patients. However, these EEG features are non-specific as they indicate diffuse cerebral dysfunction of different etiologies [12].

The measurement of protein 14-3-3 in cerebrospinal fluid is an important low-invasive technique. It showed a high sensitivity of up to 90%, but its specificity for prion diseases is lacking. Also, the 14-3-3 protein levels might not be elevated during the disease [13]. Nevertheless, the 14-3-3 protein is included as one of the diagnostic criteria for sporadic CJD [14].

The differential diagnosis of CJD is a wide spectrum of diseases that require appropriate workup. These comprise vascular neurodegenerative, autoimmune, infectious, and thromboembolic disorders, besides other conditions such as malignant metastatic, iatrogenic, toxic, or metabolic encephalopathies which typically result in progressive cognitive impairment [15]. Definitive and early diagnosis becoming possible using the real-time quaking-induced conversion (RT-QuIC) seeding assay, which detects minute amounts of the disease-specific pathologic prion protein in cerebrospinal fluid or olfactory mucosa samples [16].

Sporadic CJD is the most common form of the disease and may arise due to random mutation or post-translational modification of the PrP gene. Familial CJD, on the other hand, is linked to germline mutations of the PrP gene. Iatrogenic CJD is the transmissible form of the disease that can be acquired from medical procedures such as growth hormone treatment from pooled pituitary glands, cadaveric dura mater grafts, or corneal transplantation [2].

It is worth mentioning that the initial manifestations in the present case started after contracting COVID-19 pneumonia. We could not identify any causative link between the COVID-19 infection and CJD, however, COVID-19 is known to cause neurodegeneration and it has been proposed that COVID-19 can initiate or accelerate sporadic CJD [17]. This might be due to the COVID-19-associated systemic inflammatory response that can initiate prion misfolding. Furthermore, it has been suggested that coronavirus spike protein (S) hastens proteinopathic seeding of pathogenic tau aggregates, or it causes increased cytosolic spread of prion and tau aggregates [18]. Some case reports support the possible relationship between sporadic CJD and COVID-19 infection. Coincidence of the CJD symptoms with COVID-19 infection has been described in a 60-year-old male [19] and a 70-year-old woman [17]. Bernardini et al. [20] also defined a 40-year-old male who developed CJD manifestations two months after recovery from a mild COVID-19 infection. 

During the admission of this patient to the Neurology Department, she was on supportive therapy, and she was stable. After a hospital stay for one month, she was discharged on psychosocial support. The mainstay of the management of CJD is supportive care, and it has no definitive treatment. Most trial drugs for CJD have not demonstrated significant efficacy in increasing survival. Psychosocial support and supportive care may improve patients' quality of life [21]. 

One of the limitations of this study is that the diagnostic data for the case was incomplete. Certain crucial information was unavailable in the hospital records, which may have impacted the overall assessment of the condition. This lack of comprehensive data restricts our ability to draw definitive conclusions and may limit the generalizability of the findings. 

## Conclusions

In conclusion, it is critical to consider CJD in the differential diagnosis when neuropsychological symptoms, particularly rapidly progressive cognitive decline, are observed. Early and accurate diagnosis is essential for distinguishing this untreatable condition from other treatable causes of rapidly progressive dementias, ensuring appropriate intervention strategies and contributing to future therapeutic trials. Additionally, it is important to note that COVID-19 has been explored as a potential risk factor for neurodegenerative diseases, including prion diseases like CJD. Understanding any potential associations with COVID-19 could influence early detection and management approaches, ultimately improving patient outcomes.
